# Unremitting Asthma as a Presentation of Pulmonary Nocardiosis: A Case Report

**DOI:** 10.7759/cureus.54722

**Published:** 2024-02-22

**Authors:** Sandus Khan, Aleksandra Ignatowicz, Nivedha Balaji, Christopher R Chew, Aleksandros Mihilli, Urvesh Patel

**Affiliations:** 1 Internal Medicine, Northeast Georgia Medical Center Gainesville, Gainesville, USA

**Keywords:** immunocompromised hosts, inhaled corticosteroids, severe refractory asthma, pulmonary infections, pulmonary nocardiosis

## Abstract

Severe, refractory asthma requires a combination of multiple maintenance inhalers and medications including high-dose inhaled corticosteroids and immunomodulators to achieve control of symptoms. The use of inhaled corticosteroids, however, increases the susceptibility of opportunistic bacterial infections, such as *Nocardia*, resulting in pulmonary nocardiosis. This case describes a 46-year-old patient with a history of severe, refractory asthma who presented with progressively worsening asthma exacerbation symptoms. She was treated with immunomodulators, high-dose inhaled corticosteroids and oral steroids, and several courses of antibiotics. CT imaging revealed bibasilar peri-bronchial thickening and tree-in-bud nodularity in the right lower lobe. Pulmonary cultures collected from bronchoscopy grew *Nocardia* *nova* complex. This was a rare case of persistent asthma exacerbation by* N. nova* complex bronchopulmonary infection. Broad differentials should be considered in patients with severe, refractory asthma who were previously controlled and were found to fail treatment therapies. Immunocompromised patients with chronic lung disease are at higher risk of severe infection with disseminated nocardiosis. These patients have a higher mortality and morbidity risk if early diagnosis of pulmonary nocardiosis does not occur.

## Introduction

*Nocardia* is an opportunistic actinomycete found in ubiquitous environments and easily transmissible through inhalation or direct skin inoculation. The bacteria primarily affect up to 30% of immunocompetent patients who will more frequently have cutaneous involvement and approximately 60% of immunocompromised hosts who are more likely to suffer from pulmonary involvement [1,2]. More severe pulmonary disease states transpire in immunocompromised patients such as those on high-dose oral or inhaled steroids, transplant patients, patients receiving chemotherapy, and those with autoimmune diseases, chronic lung disease, and any condition with a cell-mediated immunity deficiency [3]. Patients with chronic structural lung diseases include those with bronchiectasis, interstitial lung disease, asthma, and most commonly chronic obstructive pulmonary disease [4]. 

Pulmonary nocardiosis is more frequently observed as the lungs are the most affected site second to skin involvement. There is an increased risk of mortality with dissemination to other organ systems, especially the central nervous system. This prompts for early identification and treatment [5,6]. Early diagnosis is difficult as the clinical presentation and radiographic findings can be nonspecific, often mimicking other disease processes and malignancies [7]. Prolonged duration of respiratory symptoms of a productive cough and dyspnea may be one of the only systemic findings prior to further workup. 

Patients with severe, refractory asthma require an extensive regimen of high-dose inhaled corticosteroids (ICS) with or without oral steroids, controller medications, and immunomodulators to obtain relative symptom control. In cases where patients are adherent to their appropriate regimen and have worsening symptoms, an underlying infection should be considered as the etiology for new onset prolonged and treatment-resistant exacerbations. This case presents a rare case of severe, refractory asthma exacerbation from pulmonary nocardiosis with one of the most common pathogenic *Nocardia* species, *Nocardia nova* complex. 

## Case presentation

A 46-year-old female with a history of severe, refractory asthma and recent coronavirus disease 2019 (COVID-19) infection presented with a five-month history of persistent chest congestion, wheezing, a productive cough, pleuritic chest pain, worsening exercise intolerance, and intermittent subjective fevers. The patient reported worsening asthmatic symptoms despite being on maximum maintenance therapy for asthma including Zafirlukast, fluticasone-umeclidinium-vilanterol inhaler, benralizumab, fluticasone inhaler, and as needed albuterol inhaler. The patient completed two rounds of oral antibiotics with doxycycline and cefdinir, as well as several courses of oral prednisone without improvement in symptoms. 

Vitals were all within normal limits. On evaluation, diffuse wheezing was heard throughout all lung fields. No rales or rhonchi were appreciated. The remainder of the physical exam was within normal limits. Pulmonary function tests (PFT) were indicative of large and small airway obstructions with small airway reversibility worse than previous PFTs. 

**Figure 1:**
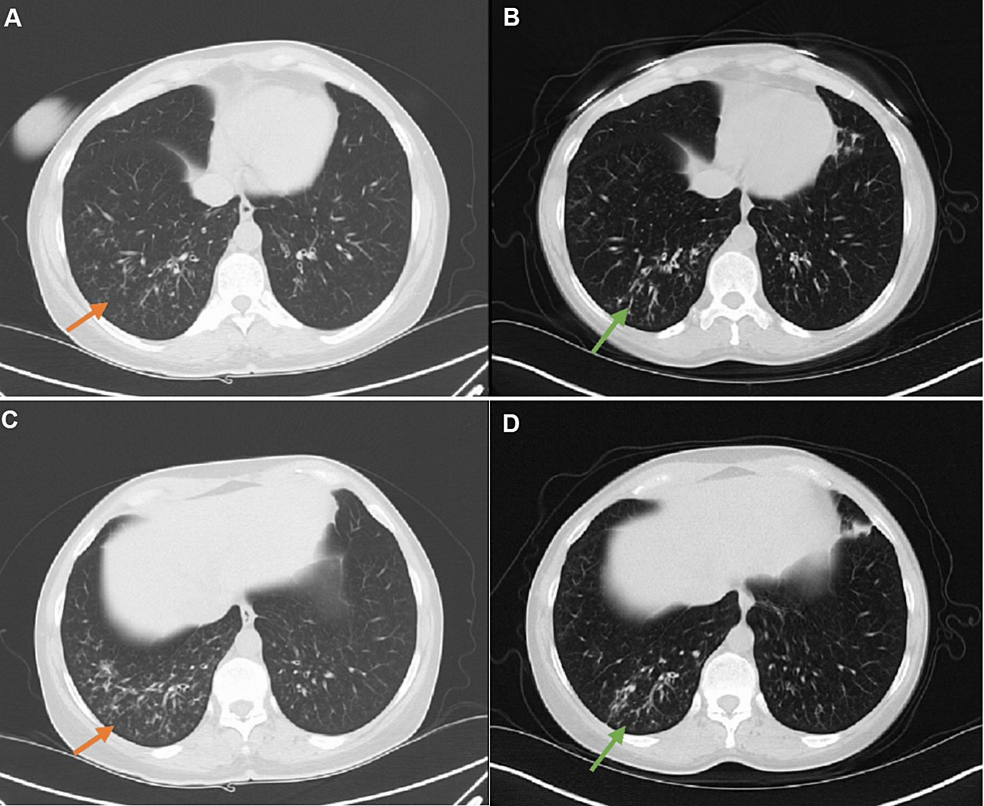
Regression of pulmonary nocardiosis during the treatment course from May 2022 to December 2022. (A, C) CT chest from May 2022 revealed mild linear opacities mostly in the right lower lobe; (B, D) Follow-up CT chest in December 2022 after seven months of treatment with TMP-SMX revealed bibasilar peribronchial thickening and tree-in-bud nodularity slightly increased in the right lower lobe and decreased in the left lower lobe. TMP/SMX: trimethoprim/sulfamethoxazole

Chest x-rays showed left lower lobe airspace disease consistent with pneumonia. CT imaging of the chest (Figure 1) demonstrated mild to moderate linear nodular opacities in the right lower lobe. Sputum cultures collected initially did not grow organisms. The patient was later referred to pulmonology and underwent bronchoscopy with bronchial alveolar lavage (BAL). The bronchoscopy revealed a large amount of thick and gelatinous secretions in her airways. The respiratory cultures from the BAL grew *N. nova* complex. 

The patient was referred to Infectious Disease and started on trimethoprim/sulfamethoxazole (TMP-SMX) for the treatment of pulmonary nocardiosis. Serial CT imaging of the chest revealed persistent bibasilar peri-bronchial thickening and tree-in-bud nodularity that was slightly increased in the right lower lobe with new left upper lobe ground glass opacities. There were no cavitary lesions, consolidations, or pleural effusions noted. MRI of the brain was also ordered to rule out disseminated nocardiosis and central nervous system involvement. Benralizumab was held until the patient completed the six-month course of TMP-SMX. While receiving TMP-SMX, the patient’s asthma symptoms and PFTs improved; however, the TMP-SMX course was prolonged to a 12-month course due to persistent complaints of a productive cough and abnormal CT chest findings. 

## Discussion

Severe, refractory asthma is characterized as persistent asthma symptoms despite maximizing maintenance therapy with high-dose ICS-long-acting β-agonist (LABAs), and appropriate management of co-morbidities. High-dose steroids are crucial in this patient population in that the airway smooth muscle cells are less responsive to corticosteroids compared to patients with mild or moderate asthma. Patients will also experience worsening symptoms with a decreased dosage of ICS [8]. These patients often require dual therapy with prolonged oral corticosteroids in addition to the high dose ICS, multiple controller agents including LABA, short-acting β-agonist (SABA), theophylline, or leukotriene antagonists. Many patients remain symptomatic and require adjuvant therapy with immunomodulators such as IgE, IL-4 receptor, and IL-5Ra inhibitors [9]. The treatment regimen raises a concern for immunosuppression with the use of corticosteroids and immunomodulators in combination with airway epithelial dysfunction predisposing to acute pulmonary infections. 

Several theories exist proposing the mechanism of pneumonia and opportunistic bacterial infections. The chronic inflammation associated with asthma induces goblet cell hyperplasia and increased mucin production resulting in impaired clearance of both colonized and pathogenic bacteria in the lower respiratory tract [10]. Furthermore, ICS alters the host mucosal defense system allowing for bacterial infiltration; however, these studies were supported in chronic obstructive pulmonary disease (COPD) patients [11]. Although there has not been a significant association between pulmonary infections with immunomodulator use, studies have shown a dose-dependent relationship with ICS, specifically with fluticasone, resulting in an increased susceptibility to pneumonia [8,12-14]. 

Multiple studies compared the use of fluticasone versus budesonide in the increased risk of community-acquired pneumonia. There has been controversial data indicating a protective effect with the use of budesonide in preventing pneumonia or a lack of association between the two [8,11,14,15]. Considering that high-dose ICS is essential for the treatment of severe, refractory asthma, pneumonia can be suspected in patients treated with high-dose fluticasone as our patient was. In addition, patients with recent viral infections are at risk of damage to the respiratory barrier with concurrent immune system dysfunction further creating conditions of opportunistic bacterial or fungal infections [16]. 

Immunocompromised patients or those with underlying structural lung disease and concurrent oral or inhaled corticosteroids use at risk of developing invasive nocardiosis [17]. Pulmonary nocardiosis is one of the most common sites of infection since inhalation is a primary route of entry for *Nocardia*. Asthma patients with impaired airway clearance and immune dysfunction from steroid use have an increased chance of* Nocardia* colonization with progression to pulmonary nocardiosis [17,18]. The prognosis of pulmonary nocardiosis is dependent on prompt diagnosis; however, initial presentation can be non-specific or with delayed symptom presentation. Radiographic studies can mimic other pulmonary diseases with findings of consolidations, pulmonary nodules, cavitary lesions, interstitial patterns, pleural effusions, and even chest wall extensions [1,3,6]. Diagnosis may require invasive procedures including bronchoscopy with BAL, lung biopsy, or needle aspiration as sputum samples are often inconclusive [18]. Once a diagnosis is established, the duration of treatment is expected for 6-12 months with either monotherapy with sulfonamides, TMP-SMX, or combination therapy by adding cotrimoxazole, linezolid, amikacin, cephalosporins, or carbapenems [19]. Pulmonary involvement may require combination therapy according to the severity of the disease and concerns of antibiotic resistance to prevent relapses [7]. In several studies, patients were treated with TMP-SMX primarily, and carbapenems were added on in severe pulmonary disease and with dissemination [2,5]. 

With the rarity of *Nocardia* infections, there is a lack of formal guideline-directed treatment compared to other infections [2]. Although there are multiple studies and case reports published, there is a lack of clinical trials to determine optimal therapies and inconsistency with the in vitro and in vivo antibiotic susceptibilities [20]. Multiple studies reported treatment with sulfonamides, although antibiotic and anti-microbial susceptibility testing is recommended to identify specific *Nocardia* strains and determine susceptibility patterns. Various *Nocardia* species have different resistance patterns, particularly with commonly used TMP-SMX [20]. Consequently, antibiotic treatment is prolonged or relapse with disease progression has been described in certain cases [20]. Further studies are needed to determine primary treatments for immunocompromised patients with nocardiosis. 

It is to be noted that the patient in the current report was diagnosed with COVID-19 several months prior to diagnosis with pulmonary nocardiosis. There have multiple documented cases of nocardiosis during or after COVID-19 infection in patients with risk factors predisposing them to a *Nocardia* infection after steroid use. However, these patients experienced a rapid onset of symptoms or were diagnosed with *Nocardia* within 5-50 days with concurrent ICS use during their hospitalization [20]. The patients were likely colonized with *Nocardia *species prior to viral infection and the use of steroids [16]. The combination of viral infection with additional immunosuppression from chronic steroid use provided an ideal environment for a superimposed *Nocardia *infection [16]. However, our patient was already treated with long-term oral and inhaled steroids at the time of her COVID-19 diagnosis. She completed a course of casirivimab-imdevimab and oral prednisone taper in (no listing of months, days, years) with a resolution of her sinus symptoms. Her worsening asthma symptoms appeared two to three months period after her COVID-19 infection. The patient’s imaging at the time of the COVID-19 infection and follow-up imaging did not show signs of lower lobe infiltration observed with the *Nocardia* infection until months later. Although the COVID-19 infection was a likely risk factor predisposing to the *Nocardia* infection, we believe that the patient’s pulmonary nocardiosis was related to persistent high-dose ICS use. 

## Conclusions

Immunocompromised patients are at risk for invasive nocardiosis and dissemination to other organ systems as the clinical diagnosis of pulmonary nocardiosis can be challenging. Oftentimes, patients are suspected to have community-acquired pneumonia, cavitary pneumonia, or mycosal infections according to non-specific respiratory symptoms, and imagining findings can be misleading. At the same time, the lack of timely diagnosis and treatment can result in dissemination and an overall poor prognosis. Patients with severe, refractory asthma are at risk of pulmonary nocardiosis with the concurrent factors of use of high-dose steroids and structural lung disease. 

## References

[1] Kandi V (2015). Human Nocardia infections: a review of pulmonary nocardiosis. Cureus.

[2] Oliveira Cabrita BM, Correia S, Jordão S, Correia de Abreu R, Alves V, Seabra B, Ferreira J (2020). Pulmonary nocardiosis: a single center study. Respir Med Case Rep.

[3] Ambrosioni J, Lew D, Garbino J (2010). Nocardiosis: updated clinical review and experience at a tertiary center. Infection.

[4] Aggarwal D, Garg K, Chander J, Saini V, Janmeja AK (2015). Pulmonary nocardiosis revisited: a case series. Lung India.

[5] Steinbrink J, Leavens J, Kauffman CA, Miceli MH (2018). Manifestations and outcomes of nocardia infections: comparison of immunocompromised and nonimmunocompromised adult patients. Medicine (Baltimore).

[6] Kancherla R, Ramanathan RP, Appalaraju B, Rajagopala S (2019). Pulmonary nocardiosis presenting as exacerbation of chronic pulmonary disease. Indian J Crit Care Med.

[7] Wilson JW (2012). Nocardiosis: updates and clinical overview. Mayo Clin Proc.

[8] Kim MH, Rhee CK, Shim JS (2019). Inhaled corticosteroids in asthma and the risk of pneumonia. Allergy Asthma Immunol Res.

[9] Menzella F, Bertolini F, Biava M, Galeone C, Scelfo C, Caminati M (2018). Severe refractory asthma: current treatment options and ongoing research. Drugs Context.

[10] Lee YJ, Park YB (2023). Inhaled corticosteroids is not associated with the risk of pneumonia in asthma. Tuberc Respir Dis (Seoul).

[11] O'Byrne PM, Pedersen S, Carlsson LG (2011). Risks of pneumonia in patients with asthma taking inhaled corticosteroids. Am J Respir Crit Care Med.

[12] Htun ZM, Aldawudi I, Katwal PC, Jirjees S, Khan S (2020). Inhaled corticosteroids as an associated risk factor for asthmatic pneumonia: a literature review. Cureus.

[13] McKeever T, Harrison TW, Hubbard R, Shaw D (2013). Inhaled corticosteroids and the risk of pneumonia in people with asthma: a case-control study. Chest.

[14] Qian CJ, Coulombe J, Suissa S, Ernst P (2017). Pneumonia risk in asthma patients using inhaled corticosteroids: a quasi-cohort study. Br J Clin Pharmacol.

[15] Sin DD, Tashkin D, Zhang X (2009). Budesonide and the risk of pneumonia: a meta-analysis of individual patient data. Lancet (London, England).

[16] Stamos DB, Barajas-Ochoa A, Raybould JE (2023). Nocardia pseudobrasiliensis co-infection in SARS-CoV-2 patients. Emerg Infect Dis.

[17] Kumar A, Hegde M, Padyana M (2017). Pulmonary nocardiosis: under-diagnosed respiratory opportunistic infection - a case report. Radiol Infect Dis.

[18] Singh I, West FM, Sanders A, Hartman B, Zappetti D (2015). Pulmonary Nocardiosis in the immunocompetent host: case series. Case Rep Pulmonol.

[19] Lafont E, Conan PL, Rodriguez-Nava V, Lebeaux D (2020). Invasive nocardiosis: disease presentation, diagnosis and treatment - old questions, new answers?. Infect Drug Resist.

[20] Laplace M, Flamand T, Ion C (2022). Pulmonary nocardiosis as an opportunistic infection in COVID-19. Eur J Case Rep Intern Med.

